# Optimization and Validation of a Headspace Solid-Phase Microextraction with Comprehensive Two-Dimensional Gas Chromatography Time-of-Flight Mass Spectrometric Detection for Quantification of Trace Aroma Compounds in Chinese Liquor (Baijiu)

**DOI:** 10.3390/molecules26226910

**Published:** 2021-11-16

**Authors:** Xiaoqing Mu, Jun Lu, Mengxin Gao, Changwen Li, Shuang Chen

**Affiliations:** 1 Laboratory of Brewing Microbiology and Applied Enzymology, Key Laboratory of Industrial Biotechnology of Ministry of Education, School of Biotechnology, Jiangnan University, Wuxi 214122, China; xqmu@jiangnan.edu.cn (X.M.); 6180201012@stu.jiangnan.edu.cn (M.G.); 2 Suqian Jiangnan University Institute of Industrial Technology, Suqian 223800, China; 3 Guizhou Guotai Liquor Group Co., Ltd., Renhuai 564500, China; lujun_0316@hotmail.com (J.L.); licw@tasly.com (C.L.)

**Keywords:** GC×GC-TOFMS, trace aroma compounds, quantitative analysis, Chinese liquor (Baijiu)

## Abstract

The detection of trace aroma compounds in samples with complex matrices such as Chinese liquor (Baijiu) requires a combination of several methods, which makes the analysis process very complicated. Therefore, a headspace solid-phase microextraction (HS-SPME) method coupled with two-dimensional gas chromatography time-of-flight mass spectrometry (GC×GC-TOFMS) was developed for the quantitation of a large number of trace compounds in Baijiu. Optimization of extraction conditions via a series of experiments revealed that dilution of the alcohol content of 8 mL of Baijiu to 5%, followed by the addition of 3.0 g of NaCl and subsequent SPME extraction with DVB/CAR/PDMS fiber coating over 45 min at 45 °C was the most suitable. To check the matrix effects, various model Baijiu matrices were investigated in detail. The quantitative method was established through an optimized model synthetic solution, which can identify 119 aroma compounds (esters, alcohols, fatty acids, aldehydes and ketones, furans, pyrazines, sulfur compounds, phenols, terpenes, and lactones) in the Baijiu sample. The developed procedure provided high recovery (86.79–117.94%), good repeatability (relative standard deviation < 9.93%), high linearity (*R*^2^ > 0.99), and lower detection limits than reported methods. The method was successfully applied to study the composition of volatile compounds in different types of Baijiu. This research indicated that the optimized HS-SPME–GC×GC-TOFMS method was a valid and accurate procedure for the simultaneous determination of different types of trace compounds in Baijiu. This developed method will allow an improved analysis of other samples with complex matrices.

## 1. Introduction

Aroma is an important feature of distilled alcoholic beverages, which can directly affect the definition of product quality, the control of safety, and consumer choice [1]. Although ethanol and water are major constituents of distilled alcoholic beverages, several hundreds of compounds from different chemical classes majorly contribute to their aroma profile [2,3,4]. The contents of these compounds are very low, but they have an important influence on the aroma of distilled alcoholic beverages due to their lower sensory perception thresholds [5]. The identification and analysis of these aroma-active compounds in distilled alcoholic beverages have been the basis and focus of aroma research [6].

More than 1000 volatile compounds have been identified in different distilled alcoholic beverages [7]. Among them, the aroma compounds are very complex with different types and properties and are present in variable concentrations ranging from less than 1 μg/L to greater than 1 g/L [8,9]. For some compounds with a very low response on mass spectrometry, if one-dimensional gas chromatography (1-D GC) has been used to quantify them, it is usually necessary to combine a variety of extraction methods, possibly coupled with multiple detectors, to analyze the same sample, which causes the analysis process to be very complicated [7]. For example, the identification of sulfur and nitrogen compounds has great advantages using a flame photometric detector (FPD) and nitrogen phosphorus detector, whereas other compounds are identified using mass spectrometry (MS) [10]. To overcome these drawbacks, comprehensive one-dimensional gas chromatography (2-D GC) was developed that offers substantial advantages over conventional 1-D GC due to its high sensitivity and chromatographic resolution [11]. Two-dimensional GC (GC×GC) allows spectra deconvolution of co-eluted peaks, which makes it a useful technique for the separation and identification of trace aroma-active compounds in complex samples, and it can simultaneously identify different classes of compounds [12].

Microextraction sample preparation techniques are currently the methods of choice to perform analytical determination [13]. Headspace solid-phase microextraction (HS-SPME) has been frequently used for analysis of volatiles. However, a drawback of such a generic HS-SPME method is that it is greatly affected by matrix effects and extraction conditions. Because of the competitive adsorption caused by the limited adsorption materials, the analyte peak may be covered [14]. In view of the excellent performance of GC×GC in the separation of trace aroma components, whose concentrations are less than 1 mg/L [11], HS-SPME-2-D GC was expected to provide more comprehensive and precise chemical information in distilled alcoholic beverages, but studies using this detection technique mainly focused on qualitative applications or the quantitative detection of certain compounds [11,12,15]. Competitive adsorption is more obvious because of the higher sensitivity of 2-D GC, but the quantitative analysis with 2-D GC is not as easy as with 1-D GC [16]. To identify the quantitative results accurately, the HS-SPME parameters need to be optimized according to the application.

Baijiu, unique to China, is one of the oldest distilled alcoholic beverages, and more than 1000 volatile compounds have been identified in it [17]. Like other distilled alcoholic beverages, Baijiu has the characteristics of high ethanol content (38–65% *vol*/*vol*), numerous components, and a large concentration span [18]. With the development of research on aroma compounds in Baijiu, the study of important trace components has become the focus [19]. Components such as geosmin, β-damascene, and furfuryl mercaptan have a great influence on the flavor of Baijiu [10,20], but there are challenges in the detection of these compounds. The objective of this study was to optimize an analytical procedure based on HS-SPME in combination with GC×GC to quantify the trace levels of aroma compounds. The effect of different parameters on the extraction efficiency of compounds with a diverse range of chemical classes was studied using HS-SPME, with special attention paid to the optimization of sample alcohol dilution. Based on the study and diminution of matrix effects, an accurate method to quantify the aroma volatiles in Baijiu was developed and validated. This method and the results from its optimization provided a reference for quantifying trace compounds in samples with complex matrices.

## 2. Materials and Methods

### 2.1. Chemicals and Reagents

The standards had purity above 98% in all cases. One hundred and twenty-one volatile compounds were obtained from Sigma-Aldrich Co., Ltd. (Shanghai, China), J&K Scientific Co., Ltd. (Shanghai, China), and Alfa Aesar (Tianjin, China). The following internal standards (ISs) were purchased from Sigma-Aldrich Co., Ltd. (Shanghai, China): 2,2-dimethylpropanoic acid, l-menthol, 2-octanol, *β*-phenethyl acetate-d_3_, *n*-hexyl-d_13_-alcohol, and 2-methoxy-d_3_-phenol. Straight-chain alkanes (C_6_–C_28_) purchased from Sigma-Aldrich were employed for the determination of linear retention indices (RIs). HPLC-grade ethanol was purchased from J&K Scientific. Lactic acid and sodium chloride (AR Grade) were purchased from Sinopharm Chemical Reagent Co., Ltd. (Shanghai, China). Ultrapure water was obtained from a Milli-Q water purification system (Millipore, Bedford, MA, USA).

### 2.2. Samples

#### 2.2.1. Baijiu Samples

Four samples of commercial Baijiu were used in this study: Fenjiu (53% ethanol by volume, Fenjiu Group Co. Ltd., Shanxi, China), Wuliangye (52% ethanol by volume, Wuliangye Group Co. Ltd., Sichuan, China), and Guotai (53% ethanol by volume, Guotai Liquor Co. Ltd., Guizhou, China). These samples were purchased from a local store and stored away from light at ambient temperature before analysis. Guotai was used for developing and validating the method.

#### 2.2.2. Model Synthetic Solution

A model synthetic solution was used for the method validation. The percentage of ethanol and pH value of the synthetic solution were 50% (*v*/*v*) and 3.5, respectively, which reproduced the properties of the Baijiu studied. To generate a matrix identical to the real Baijiu, the synthetic Baijiu contained 12 standard compounds that are the major volatiles of Baijiu, and their concentrations are similar to those in real Baijiu. They are ethyl acetate 2000 mg/L, ethyl hexanoate 1000 mg/L, ethyl butyrate 500 mg/L, ethyl lactate 700 mg/L, acetic acid 400 mg/L, butyric acid 100 mg/L, caproic acid 100 mg/L, lactic acid 100 mg/L, isoamyl alcohol 1000 mg/L, butanol 150 mg/L, *n*-propanol 100 mg/L, and acetal 500 mg/L. The solution was stored at 4 °C.

### 2.3. Optimization of HS-SPME Parameters

**Figure 1:**
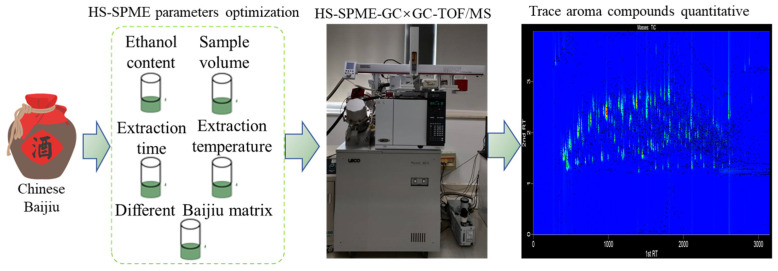
Schematic diagram of the determination of volatile compounds via HS-SPME-GC×GC-TOFMS.

The optimization procedure involved the selection of those experimental parameters that were important for the SPME extraction efficiency, and the peak areas obtained via GC×GC–TOFMS were used to evaluate the extraction efficiency [21]. To obtain the HS-SPME procedure with a maximum response area of the detected peak for extraction of compounds from Baijiu samples, the influence of sample dilution (0.5, 1, 2, 5, 10, and 15% vol), sample volume (1, 2, 4, 5, 6, and 8 mL), extraction time (15, 30, 45, 60, and 75 min), and extraction temperature (35, 40, 45, 50, 55, and 60 °C) were studied considering the high level of alcohol in the samples (Figure 1). Ultrapure water was used to dilute the Baijiu sample to make a solution with the desired ethanol concentration. The solution was saturated with NaCl, and different volumes of the diluted Baijiu sample were added to a 20 mL headspace glass vial. To create calibration curves and quantitation of volatile compounds in the Baijiu sample, 20 μL of the ISs mixture (final concentration: 2,2-dimethylpropanoic acid, 1197.55 μg/L; L-menthol, 700.19 μg/L; 2-octanol, 69.84 μg/L; *β*-phenethyl acetate-d_3_, 20.12 μg/L; *n*-hexyl-d_13_-alcohol, 200.05 μg/L; and 2-methoxy-d_3_-phenol, 80.14 μg/L) was added during sample preparation. After that, the vial was sealed with a PTFE/silicone septum and a screw top.

All runs were carried out with a 2 cm divinylbenzene/carbon wide range/polydimethylsiloxane (DVB/CAR/PDMS) 50/30 μm fiber obtained from Supelco (Bellefonte, PA, USA). DVB/CAR/PDMS SPME fibers were previously demonstrated to be suitable for analysis of trace volatile and semi-volatile compounds in Baijiu and were consequently used during this study [22]. The HS-SPME procedure was performed using a MPS autosampler (Gerstel Inc., Mulheim, Ruhr, Germany) and ChromaTOF software (LECO Corp., version 4.61.1). Samples were incubated for 5 min at the extraction temperature under continuous agitation (400 rpm) for equilibration, and then the fiber was exposed to the headspace. The desorption in the GC×GC injector was performed for 5 min at a temperature of 250 °C in the splitless mode. Each sample was analyzed in triplicate.

### 2.4. GC×GC-TOFMS Conditions

A LECO Pegasus^®^ 4D GC×GC-TOFMS (LECO Corp., St. Joseph, MI, USA) was used for all experiments. This instrument consisted of an Agilent 7890B GC (Agilent Technologies, Palo Alto, CA, USA), equipped with a liquid nitrogen-based quad-jet dual-stage cryogenic modulator (LECO Corp., St. Joseph, MI, USA), and a secondary oven, coupled with Pegasus 4D TOFMS (LECO Corp., St. Joseph, MI, USA), was applied for the analysis. The primary column was a 60 m × 0.25 mm × 0.25 μm DB-FFAP (Agilent Technologies, Palo Alto, CA, USA) connected in series with a 1.5 m × 0.25 mm × 0.25 μm Rxi-17Sil MS secondary column (Restek, Bellefonte, PA, USA).

GC×GC-TOFMS conditions that were previously reported [23] were used. The sample extract was injected in splitless mode at an injector temperature of 250 °C. The separation was performed using the following optimized temperature program for the primary oven: held at 45 °C for 3 min, increased at 4 °C/min to 150 °C, then held for 2 min, raised at 6 °C/min to 200 °C, followed by an increase at 10 °C/min to 230 °C, and held for 10 min. The secondary oven temperature was operated at a constant offset of 5 °C relative to the primary one. The carrier gas was high purity helium (≥99.999%), at a constant flow rate of 1 mL/min. The modulator was offset by +20 °C in relation to the primary oven. A modulation period of 4 s (alternating 0.8 s hot and 1.2 s cold) was used.

The MS transfer line and the ion source were maintained at 240 °C and 230 °C, respectively. The TOFMS detector was operated in the electron impact ionization energy mode at 70 eV with the electron voltage set at 1430 V. The data were collected over a mass range of 35–400 amu at an acquisition rate of 100 spectra/s following no acquisition delay. Data acquisition and analysis were performed using LECO ChromaTOF software.

### 2.5. Processing and Analysis of Chromatographic Data

The chromatographic data were processed and aligned using spectral deconvolution algorithms implemented in the ChromaTOF software (LECO Corp., version 4.61.1). Automated peak finding and spectral deconvolution with a baseline offset of 0.5 and a signal-to-noise ratio of 100 were used. These conditions allowed the unique identification of each chromatographic feature in the context of high dynamic range samples [24]. For the alignment of peaks across chromatograms, maximum one- and two-dimension retention time deviations were set at 12 s and 0.2 s, respectively.

Compounds were identified based on the comparison of their MS and RIs with those of pure standards under the same chromatographic conditions described for the samples. All compounds of interest tentatively assigned by the ChromaTOF software were manually assessed with respect to the mass spectra match and the assigned unique mass that was used for quantification. The MS with two commercial libraries (NIST 2014 and the Weliy9 databases) match factor, similarity >700, was used to decide whether a peak was correctly identified. It was determined to be an appropriate value based on a previous nontargeted study on volatile organic compound mixtures [25]. GC×GC analysis of C_6_–C_28_ straight chain alkanes was performed to determine one-dimensional linear retention indices (RIcal) for each compound. In addition to the comparisons with the RIs of pure standards, the RIcal was also compared with the RIs reported in the literature and NIST library (RIlit). A maximum deviation of 30 between the RIcal and RIlit values was used as the criterion.

### 2.6. Method Validation

#### 2.6.1. Calibration and Detection Limits

Calibration curves were created for the quantification of volatile compounds using the optimized HS-SPME-GC×GC-TOFMS conditions. Individual standard stock solutions were mixed in different categories and then diluted with the solution mentioned in Section 2.2.2 to a serial concentration to set up the calibration curve. The sample preparation method and IS addition amount used for calibration were the same as those used for the analysis of Baijiu samples. The linear ranges of the method were analyzed by creating calibration curves using different concentration levels of a model synthetic solution. The linearity of each compound was determined via evaluation of the regression curves (ratio between the area of the chromatographic peak of the standard and the area of the IS against the concentration ratio) and was expressed using the coefficient of determination (*R*^2^). The limits of detection (LOD) and quantitation (LOQ) were determined from the calibration curves’ data. The LOD was defined as the lowest concentration of the calibration curve based on a signal-to-noise ratio of 3 and the LOQ on a signal-to-noise ratio of 10. All analyses were performed in triplicate.

#### 2.6.2. Precision and Accuracy

A sample of Guotai was spiked with three concentrations of standard solution for precision and accuracy tests according to the guide. The intraday precision was evaluated using GC×GC-TOFMS analysis of the same sample three times on the same day. The interday precision was determined by repeating the intraday precision study on three different days. All analyses were performed in triplicate and the precision was calculated using the relative standard deviation (RSD, %) of those values. The recovery was determined through the calculation of the deviation percent between the calculated value and the nominal value.

## 3. Results and Discussion

### 3.1. Optimization of the HS-SPME Methods

**Figure 2:**
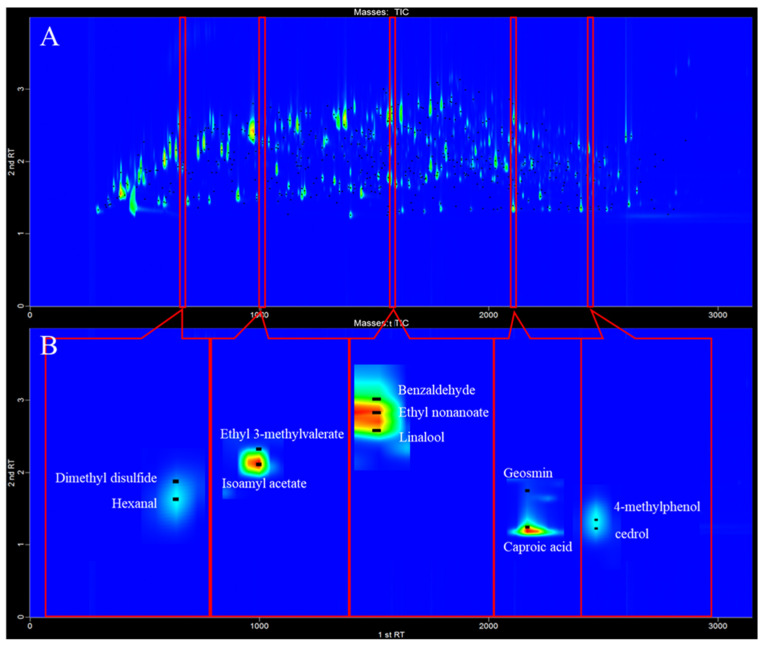
Analytical ion chromatogram contour plot for the SPME–GC×GC–TOFMS analysis of the Guotai sample: (**A**) complete two-dimensional contour plot and (**B**) detailed portions of the contour plot.

Compared with 1-D GC, 2-D GC provides improved analyte peak capacity, along with reducing the problem that chromatographic peaks are masked by the matrix. Figure 2 illustrates a two-dimensional contour plot obtained for the Guotai sample. The compounds displayed in this figure could not have been separated using conventional 1-D-GC methods, especially some trace compounds that may be masked by high-content compounds. To detect trace compounds in Baijiu comprehensively, the parameters of the HS-SPME method need to be optimized. This was achieved using 119 representative trace compounds naturally present in Baijiu (rather than the model synthetic solution, spiked with standards). These representative trace compounds belonged to quite different chemical classes, which also had an important effect on the aroma of Baijiu.

Among the parameters affecting the extraction efficiency, most were set to the same values in the various SPME methods published. For example, it is common to saturate with sodium chloride (NaCl) to promote aroma release and use magnetic stirring [26]. However, for a complex matrix such as Baijiu and a stronger response of the combined detector, some critical parameters, such as sample dilution, sample volume, extraction time, and extraction temperature, needed to be re-optimized. A compromise solution of SPME optimization should always be taken into consideration and each experiment was performed under the best optimization parameters for the previous experiment. The evaluation index of the optimization results is not the total peak area of all compounds, but they are classified and compared to avoid the wrong choice of optimal conditions because the peak area of a certain type of compound is too large.

#### 3.1.1. Effect of Sample Dilution

**Figure 3:**
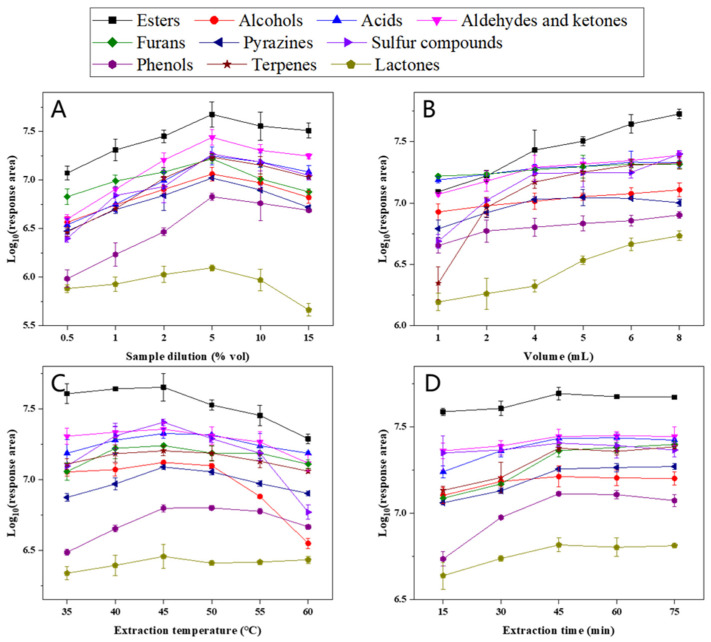
Effects of parameters on the 10 different chemical classes of compounds’ peak area: (**A**) sample dilution, (**B**) liquid volume, (**C**) extraction temperature, and (**D**) extraction time.

Because HS-SPME is an equilibrium process, when HS-SPME-GC×GC-TOFMS is used to quantify trace compounds, a problem of competitive adsorption exists. Ethanol is the major matrix constituent of Baijiu and has been reported as an important interfering volatile during HS-SPME of trace compounds [27], especially for the hydrophobic analytes, which suffered more strongly from the competition between the aqueous alcoholic solution and the fiber coating [28]. The selection of an appropriate dilution ratio may reduce matrix interferences [29], so we reduced the effect of ethanol in quantitative analysis via sample dilution. Six different diluted alcohol levels of 0.5, 1, 2, 5, 10, and 15% *v*/*v* were carried out. Figure 3A shows that the response areas of all types of trace compounds increased first and then decreased with the change of diluted alcohol content and maximum extraction efficiency at 5% alcohol. Different from the 10% alcohol optimized using HS-SPME-GC-MS [22], this may be due to GC×GC-TOFMS being more sensitive in detection and therefore more affected by competitive adsorption. Moreover, lactones are more affected by alcohol, and the peak area decreases rapidly above 5% alcohol. Because ethanol prevented the studied trace analytes from being adsorbed on the saturated fiber, the selection of an appropriate dilution of 5% *v*/*v* may reduce competitive adsorption and make the results more accurate.

#### 3.1.2. Effect of Sample Volume

Studies have shown that for higher sensitivity of HS-SPME and thus extraction yield of compounds, the sample headspace should be as small as possible [30], but there are few studies reporting optimizing SPME by adjusting the sample volume [26]. To prove the effect of volume on the extraction efficiency of trace compounds, six different sample volumes of 1, 2, 4, 5, 6, and 8 mL were used. Figure 3B shows that the response areas increased with the increase of sample volume. To the best of our knowledge, most studies chose half the volume of the headspace glass vial [31]. However, in actual analyses, 20 mL headspace vials containing up to 8 mL of liquid were used to prevent the SPME fiber from contacting the liquid, which is also the optimal filling volume within the achievable range. Therefore, the optimal volume of the sample placed in a 20 mL vial was 8 mL.

#### 3.1.3. Effect of Extraction Temperature and Time

The extraction temperature was evaluated in univariate mode at 35, 40, 45, 50, 55, and 60 °C while keeping the other variables at their optimum value. Figure 3C indicates that the extraction efficiency of most types of trace compounds increased first and then decreased with the change of extraction temperature, but the trend of individual compounds may not be obvious. Among them, sulfur compounds and pyrazines decreased significantly above 45 °C, and alcohols decreased significantly above 50 °C. This indicated that volatile compounds that were entirely in the gaseous phase at a specific temperature will adsorb less on the fiber at a higher temperature. Moreover, at a temperature above 50 °C, the properties of some compounds will change [32]. The results of the analysis performed in triplicate indicated that the extraction temperature of 45 °C was a compromise temperature for all compounds and was used for further analysis.

As the last parameter, the extraction time was assessed using variation between 15 and 75 min. Figure 3D shows that the extraction efficiency of almost all compounds increased first and then leveled off with the change of extraction time; the same trends for HS-SPME-GC-MS were seen in previous studies [33]. However, the peak area of sulfur compounds and phenols decreased slightly after the extraction time exceeded 45 min, which could be explained by competition effects during adsorption to the fiber. Because our targets are trace compounds, the concentration of the other high content components in the headspace increased with increasing extraction time, and due to their higher affinity for the fiber, some of the target compounds may desorb from the fiber due to competition. Therefore, the procedure of 45 min, according to the optimal accuracy with time-efficient extraction, was used for further analysis.

### 3.2. Assessment of the Matrix Effects

With regard to the detection of volatile compounds, one of the challenges encountered when developing quantitative extraction methods is the influence of other matrix components; the headspace equilibrium of substances is greatly influenced by the presence of volatile compounds other than the selected substances [34]. The target analytes in our study were trace compounds with a content of less than 1 mg/L in Baijiu. However, these trace compounds were affected by competitive adsorption with high content compounds in the sample during quantification, resulting in a lower response. To compensate for such matrix effect, it was decided the IS method would be used to construct standard calibration curves to evaluate the headspace concentration of volatiles from GC peak area responses. Six ISs, including three isotopically labeled ones, were used in our study. The selection of the matrix for quantitative calibration curves played an important role in this method. It was necessary to make the response value of the target compound in the model synthetic matrix consistent with the response value of the real Baijiu sample, otherwise, it caused a large difference in peak area and inaccurate results.

**Figure 4:**
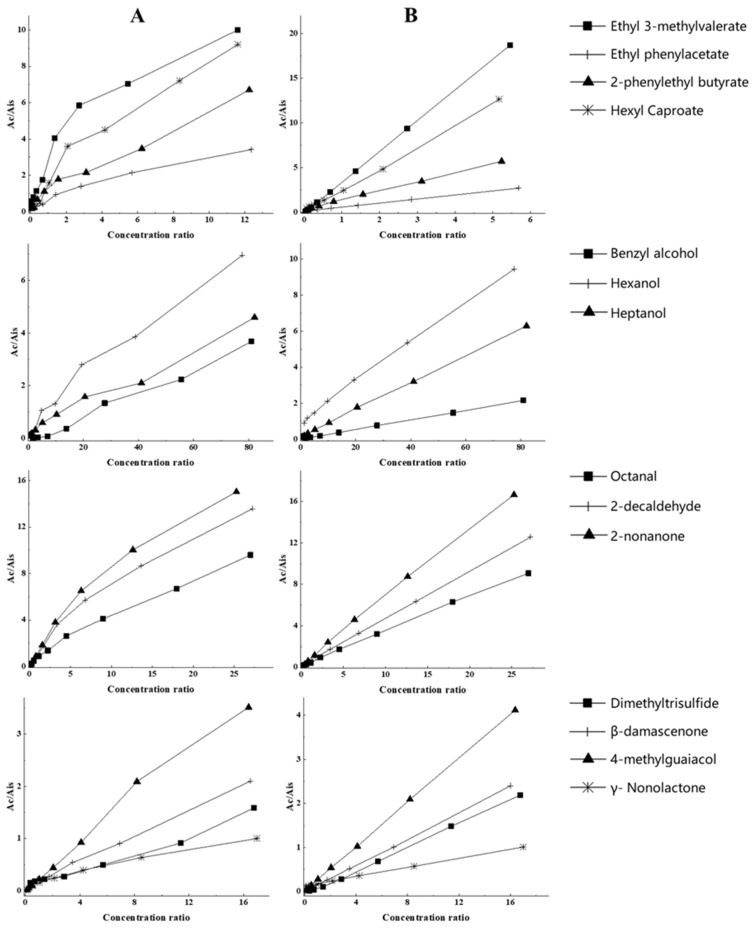
Standard curves of different chemical classes of compounds in the quantitative analytes: (**A**) SS and (**B**) SS + HCV.

To check the matrix interference in detail, several solutions of different model Baijiu matrices were analyzed using HS-SPME-GC×GC-TOFMS, and the resulting chromatographic peak areas were compared. These included 50% water/ethanol solution at pH 3.5, which is also a common model synthetic solution in the quantitative analysis of volatile compounds in Baijiu [22], referred to as SS (simple solution); 50% water/ethanol solution at pH 3.5 with some high content volatiles in Baijiu, referred to as SS + HCV; and a real Baijiu sample (50% ethanol). These matrices were spiked with the same amounts of analytes, and the final concentrations of analytes were close to those of the real Baijiu sample. Table 1 shows the relative response of the different classes of compounds in these matrices. The peak areas of analytes in SS + HCV were significantly lower than those in SS, indicating the presence of some type of competition between the interfering substances and analytes in the matrix. The chromatographic response of real Baijiu was close to the response in SS + HCV. The calibration plots of different chemical classes of trace compounds in SS and SS + HCV are shown in Figure 4. For SS, the low concentration mixed standard solution had a different trend from the high concentration mixed standard solution, which may be the reason for the inaccurate quantification of the corresponding peak overload. However, the linearity of the corresponding standard curve in SS + HCV was improved.

Therefore, in the calibration and quantification steps, we worked with the model synthetic solution described in Section 2.2.2. The application of this model synthetic solution could not only avoid matrix effects but also expand the quantitative range of trace components in quantitative analysis using HS-SPME-GC×GC-TOFMS.

### 3.3. Method Validation

The proposed method was validated and applied to determine the concentration of 119 trace volatile compounds in Baijiu using HS-SPME-GC×GC-TOFMS. The quantitative method for 26 esters, 11 alcohols, six acids, 24 aldehydes and ketones, six furans, eight pyrazines, 11 sulfur compounds, seven phenols, 16 terpenes, and four lactones was constructed using a model synthetic solution under the optimal conditions. The performance of the method regarding linearity, detection limits, LOD, LOQ, precision, and accuracy for each compound are shown in Table 2.

Good linearity could be obtained for all volatile compounds at the concentration studied, with coefficients of determination (*R*^2^) above 0.99. The developed method had good precision because all RSD values calculated for intraday precision varied between 0.14% and 9.34% and interday precision varied between 0.14% and 9.93%. Moreover, the recovery values varied from 86.79% to 111.94%, which indicated that the developed method was accurate for determining trace compounds in Baijiu.

The lowest LOD and LOQ of all compounds were for methyl nonyl ketone, 0.04 ng/L and 0.14 ng/L, respectively, and the highest LOD and LOQ were for hexanol (12.93 μg/L and 43.09 μg/L). Some of these compounds were hundreds of times lower than those reported in the literature using HS-SPME-GC-MS analysis [35,36]. For instance, the LOD of 3-octanol in this study was 6.98 ng/L, which was 27 times lower than that of HS-SPME-GC-MS, which had a LOD of 189.39 ng/L [37]. Eleven sulfur compounds were quantitatively analyzed using the optimized method, and the LOQ of 1.36 ng/L for dimethyl trisulfide was 198 times lower than the 0.27 μg/L achieved with the GC-PFPD [10]. These results indicated that this method had obvious quantitative analysis advantages compared with GC-MS and even specific element analysis instruments, and it was an effective method for quantitative analysis of trace compounds in Baijiu.

### 3.4. Analysis of Baijiu Samples

The optimized HS-SPME–GC×GC-TOFMS method was applied to different types of Baijiu samples to demonstrate its effectiveness. The mean concentration values of the 119 volatile compounds in Baijiu samples are presented in Table 3. The lowest concentration of compounds detected was γ-decalactone in the Wuliangye sample, which was only 0.28 μg/L. The 3-methylbutyraldehyde had the highest concentration in the light aroma type and soy sauce aroma type Baijiu, which was 82,260.03 μg/L in the Moutai sample. This method achieved the simultaneous quantification of different chemicals and different concentrations of compounds in a complex matrix.

The odor activity values (OAVs) of volatile compounds in different types of Baijiu samples are also presented in Table 3. The OAV was calculated by dividing the concentration by the respective reported odor threshold, which can be used to measure the aroma contribution of volatile compounds in the Baijiu samples [35]. The Fenjiu sample showed the presence of 34 volatile compounds that had an OAV >1, the Wuliangye sample showed 51 volatile compounds with an OAV >1, and the Moutai sample showed 50 volatile compounds with an OAV >1, which indicated that the odors in these media could be perceived by the human nose [38].

The ethyl esters are the most important group of yeast-synthesized aroma substances in Baijiu, which mainly produce the pleasant fruit odors. Because their concentrations are much higher than aroma thresholds, they make an important contribution to the flavor of Baijiu [39]. In addition to the reported compounds, this study also quantified some esters with a lower content in Baijiu for the first time. The content of ethyl 3-methylpentanoate and ethyl cyclohexanoate in the three Baijiu samples was only tens of μg/L. The contents of ethyl 4-methylpentanoate in Fenjiu, Wuliangye, and Moutai samples were 62.42, 622.67, and 263.38 μg/L, respectively. The aroma threshold of ethyl 4-methylpentanoate in water is 0.01 μg/L [40], and our group has measured the threshold value of 21.4 μg/L in 50% aqueous alcohol. This meant that the contents of these compounds were low, but above the aroma thresholds, so these compounds may make some contribution to the flavor of Baijiu.

Sulfur compounds also play an important role in the flavor of Baijiu, which mainly present unpleasant odors of onion and rotten cabbage. This study quantified 11 types of sulfur compounds in different Baijiu samples. The highest concentration was 3-methylthiopropanol in the Moutai sample, which was 732.65 μg/L, and the lowest concentration was methyl (2-methyl-3-furanyl) disulfide in the Fenjiu sample, which was only 0.44 μg/L. Among them, although the content of furfuryl mercaptan in soy sauce aroma type Baijiu was only 35.20 μg/L, its aroma threshold was 0.1 μg/L in 46% ethanol/water solution [10], and the calculated OAV was as high as 352, which made an important contribution to the aroma of soy sauce aroma type Baijiu.

In addition, this study also quantified many terpenes with very low content, most of which are below 50 μg/L. There were six types of terpene compounds in different types of Baijiu with OAVs greater than 1, which may contribute to its aroma. In the soy sauce aroma type Baijiu, the OAV of β-damascenone was 72.43, the OAV of eucalyptol was 64.51, and the OAV of linalool was 5.94. The aroma thresholds were all measured in 46% ethanol/water solution. These three compounds made important aroma contributions to soy sauce aroma type Baijiu. This study quantified farnesol and rosoxide for the first time in Baijiu. The aroma thresholds of these two compounds measured in water were extremely low. They may contribute to the aroma of soy sauce aroma type Baijiu.

## 4. Conclusions

In this study, the combination of HS-SPME and GC×GC-TOFMS was used for quantitative detection of trace components in Baijiu samples, and matrix interferences were investigated. We optimized a series of extraction conditions, namely, sample dilution, sample volume, extraction temperature, and time for SPME analysis of volatile compounds in Baijiu. Optimization of extraction conditions via a series of experiments revealed that dilution of the alcohol content of 8 mL of Baijiu to 5%, followed by the addition of 3.0 g of NaCl and subsequent SPME extraction with DVB/CAR/PDMS fiber coating over 45 min at 45 °C was the most suitable. We evaluated the model synthetic solution used in quantification to minimize the influence of matrix effects on samples with complex matrices such as Baijiu. A calibration curve was established, and validation was performed for the 119 trace volatile compounds that were considered the main contributors to the aroma of Baijiu. The validation studies demonstrated that the proposed method met the requirements of linearity, precision, accuracy, and sensitivity for the measurement of volatile compounds in Baijiu. The improvement of sample pretreatment methods for comprehensive 2-D-GC analyses in the future would focus on the high boiling point and strong polar compounds in Baijiu.

## Figures and Tables

**Table 1:** Comparison of peak areas of volatile compositions in various matrices.

Volatile Compound	Peak Area Percentage (%) ^a^			
	Water	SS	SS + HCV	Baijiu ^b^
**Esters**				
Phenethyl butyrate	100	90	52	48
Ethyl cinnamate	100	71	41	38
Methyl benzoate	100	98	95	88
**Alcohols**				
1-Nonanol	100	47	20	18
1-Octen-3-ol	100	70	59	58
Benzyl alcohol	100	72	35	29
**Aldehydes and ketones**				
1-Octen-3-one	100	82	56	43
E-2-heptenal	100	77	33	30
Trans-2-nonenal	100	87	47	49
**Furans**				
2-Acetylfuran	100	62	23	16
Ethyl 2-furoate	100	90	62	52
**Pyrazines**				
2,3-Dimethylpyrazine	100	52	16	13
2-Ethyl-6-methylpyrazine	100	59	25	16
2,3-Diethylpyrazine	100	68	75	69
**Sulfur compounds**				
Methyl thiobutyrate	100	60	29	18
Thiazole	100	27	15	11
Ethyl 3-methylthiopropionate	100	88	44	44
**Phenols**				
4-Methylguaiacol	100	90	74	77
4-Methylphenol	100	96	74	69
4-Ethylphenol	100	88	87	86
**Terpenes**				
Isophorone	100	90	57	52
β-Cyclocitral	100	82	77	69
Geraniol	100	83	76	71
**Lactones**				
γ-Valerolactone	100	53	30	32
γ-Butyrolactone	100	56	39	13
γ-Nononactone	100	92	81	80

a: The ratio of the peak area of volatile compounds in different matrices to the peak area in water. b: The real Baijiu (Guotai, 53% vol) was diluted to 50% vol.

**Table 2:** Liner range, coefficients of determination, limits of quantification (LOQ), limits of detection (LOD), precision, and recovery results of the proposed method.

Volatile Compounds	Linear Range (μg/L)	R^2^	LOD (ng/L)	LOQ (ng/L)	Intraday Precision (%)	Interday Precision (%)	Recovery (%)
**Esters**							
Isoamyl acetate	180.12–92,220.01	0.992	1148.12	3827.07	5.06	6.28	98.13
Ethyl phenylacetate	17.88–2289.20	0.9978	3.48	11.61	0.99	4.67	100.46
Phenethyl acetate	96.28–770.32	0.9948	10.31	34.37	1.85	0.68	90.26
Phenethyl butyrate	0.98–499.92	0.9988	1.54	5.13	3.06	1.98	105.04
Ethyl propionate	370.88–11,868.92	0.997	106.94	356.48	1.21	4.84	98.16
Isobutyl hexanoate	4.88–2497.87	0.9926	5.30	17.68	6.20	9.08	86.86
Isoamyl butyrate	6.42–408.65	0.9931	2.58	8.61	1.55	8.12	107.80
Ethyl laurate	10.27–657.14	0.9912	1.81	6.03	1.94	5.73	87.32
Butyl butyrate	2.25–1151.63	0.9971	3.31	11.04	4.39	8.28	98.52
Ethyl decanoate	32.79–4196.64	0.9944	2.37	7.89	5.34	9.04	98.73
Diethyl succinate	66.29–8485.24	0.9916	54.80	182.67	0.87	0.97	92.08
Ethyl nonanoate	43.64–2793.24	0.9918	6.78	22.59	6.66	8.15	96.24
Butyl acetate	5.93–699.58	0.9911	3.03	10.10	2.11	9.59	96.08
Ethyl 3-phenylpropionate	6.45–6609.15	0.9986	2.38	7.95	2.49	8.43	101.19
Isoamyl octanoate	8.44–1080.32	0.9943	0.64	2.13	3.76	2.48	96.47
Ethyl 4-methylpentanoate	2.93–750.04	0.9962	105.66	352.19	4.75	7.54	97.83
Ethyl cyclohexanoate	0.57–73.52	0.9973	46.24	154.13	2.57	5.00	102.98
Ethyl 2-methylpentanoate	0.20–100.01	0.9975	85.61	285.37	8.67%	7.93	92.72
Pentyl hexanoate	7.99–4091.65	0.9963	3.75	12.49	7.14	8.47	106.11
Ethyl 3-methylpentanoate	0.54–34.48	0.9972	37.44	124.81	1.06	2.09	90.60
Propyl hexanoate	93.78–12,003.71	0.9926	18.38	61.26	1.24	5.26	103.77
Hexyl hexanoate	19.52–2498.12	0.9947	5.86	19.55	7.35	8.22	104.70
Ethyl 2-methylbutyrate	11.96–6133.28	0.9971	15.43	51.44	0.69	6.26	91.50
Methyl benzoate	2.03–259.48	0.9847	3.30	11.01	4.00	1.52	96.04
Ethyl benzoate	31.77–4066.20	0.99	341.25	1137.48	2.90	1.31	98.53
Ethyl 2-methylpropionate	320.53–12,497.38	0.9882	119.84	399.47	3.16	4.34	111.53
**Alcohols**							
Phenyl alcohol	94.68–12,119.28	0.9992	68.64	228.79	0.90	7.31	102.07
Benzyl alcohol	69.48–4446.43	0.9992	199.71	665.70	2.29	7.09	103.27
Hexanol	242.69–62,128.28	0.9907	12,928.22	43,094.07	2.15	7.18	96.70
Heptanol	11.20–1434.06	0.9904	7.33	24.42	0.91	4.72	110.33
Octanol	32.25–1032.04	0.9912	19.91	66.37	8.08	5.25	100.30
Nonanol	2.93–93.69	0.9986	117.90	393.01	5.55	7.01	101.36
1-Octen-3-ol	1.56–399.10	0.9919	1.37	4.57	6.73	9.45	88.32
2-Heptanol	3.01–1540.65	0.9975	2.00	6.68	2.29	9.46	89.10
3-Octanol	19.08–305.34	0.9901	6.98	23.28	1.91	7.32	110.88
2-Nonanol	3.93–125.66	0.9961	1.57	5.23	0.99	4.60	105.82
Pentanol	411.42–26,330.92	0.9964	500.59	1668.64	2.68	1.46	110.10
**Acids**							
Pentanoic acid	177.41–11,354.34	0.9973	1372.23	4574.09	2.59	9.10	109.25
Heptanoic acid	242.35–31,020.48	0.9934	117.06	390.19	6.90	4.99	104.77
Octanoic acid	44.84–11,479.92	0.9957	41.33	137.77	1.29	8.60	109.49
Decanoic acid	39.10–5005.16	0.9911	30.57	101.90	3.88	6.02	92.30
3-Methylbutanoic acid	583.78–74,723.63	0.9982	1007.61	3358.72	8.97	9.80	93.50
4-Methylpentanoic acid	28.00–7168.97	0.9906	1404.05	4680.17	2.62	7.67	101.36
**Aldehydes and ketones**							
Decanal	13.68–7005.85	0.9986	3.55	11.84	6.07	7.34	100.18
(E)-2-Heptenal	2.44–624.05	0.9999	143.06	476.85	6.72	3.20	102.62
(E)-2-Nonenal	16.07–4113.58	0.9987	220.85	736.18	7.24	5.58	99.75
(E)-2-Octenal	1.88–962.22	0.9929	72.72	242.39	6.87	0.32	93.76
2,3-Butanedione	35.14–17,990.24	0.9926	1335.46	4451.53	5.75	1.48	90.53
3-Hydroxy-2-butanone	156.53–80,142.00	0.9967	306.35	1021.18	4.86	8.87	93.53
2-Methylpropanal	47.19–24,160.00	0.9954	525.86	1752.88	0.86	5.98	96.05
2-Methylbutanal	33.75–4320.10	0.9913	62.70	209.00	6.11	2.99	101.95
Benzaldehyde	58.21–1862.72	0.999	58.56	195.20	4.99	6.75	99.25
2-Octanone	11.22–5743.20	0.9905	13.45	44.82	5.55	4.88	86.79
Phenylacetaldehyde	103.33–13,226.36	0.9986	1004.68	3348.92	0.77	7.57	98.87
Propanal	16.71–8555.70	0.9935	292.70	975.67	1.16	4.17	99.70
Octanal	1.76–900.13	0.9926	23.65	78.84	5.02	2.94	106.08
Nonanal	5.60–2868.71	0.9943	6.83	22.77	5.72	3.64	99.36
1-Octen-3-one	0.39–199.77	0.9982	38.02	126.72	1.89	2.82	101.62
(E, Z)-2,6-Nonadienal	0.24–124.82	0.9981	33.64	112.13	1.54	3.89	105.50
3-Methylbutanal	833.01–106,624.86	0.9935	177.73	592.42	7.44	8.91	110.06
(E, E)-2,4-Hexadienal	3.90–499.25	0.9945	50.40	167.99	7.62	0.51	105.77
(E, E)-2,4-Decadienal	0.70–177.98	0.9919	15.13	50.44	0.60	0.40	90.92
(E, E)-2,4-Nonadienal	0.39–50.04	0.9924	6.14	20.46	0.82	5.47	89.37
Hexanal	34.35–8793.90	0.9993	558.22	1860.74	1.39	2.02	93.31
1,1,3-Triethoxypropane	6.88–3520.47	0.9988	6017.76	20,059.20	0.44	6.12	97.18
2-Nonanone	3.45–1765.25	0.994	1.13	3.77	0.71	7.40	91.82
Acetophenone	3.27–417.96	0.9924	8.77	29.22	1.01	4.16	92.81
**Furans**							
5-Methyl-2-acetylfuran	35.48–4541.41	0.9993	46.21	154.03	3.79	9.50	99.75
Furfuryl alcohol	11.52–5900.35	0.9897	730.23	2434.10	4.37	9.24	102.21
Furfural	200.81–51,406.86	0.9958	153.63	512.11	7.77	0.95	101.29
2-Acetylfuran	6.35–3249.79	0.9961	49.68	165.59	0.60	4.28	93.58
Ethyl 2-furoate	18.26–584.20	0.999	57.53	191.76	1.89	9.62	97.75
5-Methyl furfural	9.77–5003.88	0.9949	35.51	118.36	1.67	9.03	92.99
**Pyrazines**							
2,6-Dimethylpyrazine	42.63–21,824.65	0.9962	282.14	940.48	6.39	7.40	99.70
2-Methylpyrazine	30.53–3907.93	0.9991	1234.73	4115.76	2.05	8.89	101.51
2,3,5,6-Tetramethylpyrazine	13.79–7058.28	0.9934	222.93	743.08	4.80	4.82	102.52
2-Methyl-6-ethylpyrazine	7.82–4001.93	0.9975	78.95	263.18	3.83	9.93	97.75
2,3,5-Trimethylpyrazine	10.24–5244.50	0.995	126.96	423.18	5.84	8.64	105.46
2,3-Diethylpyrazine	1.97–503.46	0.9881	244.59	815.29	5.86	4.72	96.43
2,3-Diethyl-5-methylpyrazine	0.39–200.30	0.9986	90.86	302.88	4.26	8.88	95.33
2,3-Dimethylpyrazine	1.54–787.52	0.9898	654.62	2182.06	6.26	6.57	90.94
**Sulfur compounds**							
Methional	54.87–28,092.79	0.9989	41.47	136.85	6.04	9.62	98.64
Methyl furfuryl disulfide	5.30–403.27	0.992	1.75	5.83	4.37	6.35	95.38
Dimethyl disulfide	9.71–621.28	0.9964	571.31	1904.37	6.48	6.10	100.51
Furfuryl mercaptan	2.31–1182.16	0.9917	212.26	707.52	4.27	2.03	109.64
Ethyl 3-methylthiopropionate	1.32–674.00	0.9952	165.67	552.24	4.12	1.55	101.58
Methyl thiobutyrate	0.78–399.74	0.9981	148.20	494.00	2.57	7.30	109.68
Thiazole	1.58–810.31	0.9987	25.02	82.57	9.02	0.14	100.09
Dimethyl trisulfide	24.85–795.25	0.993	1.36	4.49	1.59	4.92	94.21
Methionol	50.58–12,949.55	0.9962	14.44	47.65	5.62	6.97	99.75
Methyl 2-methyl-3-furyl disulfide	0.20–50.10	0.9911	26.08	86.95	4.07	9.83	96.42
Methanethiol	3.91–4000.00	0.9979	34.38	114.59	8.89	2.92	101.94
**Phenols**							
4-Methylphenol	25.41–3252.20	0.9925	48.96	163.19	8.80	6.60	107.67
3-Methylphenol	0.78–199.90	0.9945	26.24	87.45	9.10	7.38	90.94
Phenol	5.08–649.68	0.9916	16.48	54.93	2.08	2.56	111.94
4-Ethylphenol	3.72–475.86	0.9987	13.17	43.88	4.71	8.70	92.53
4-Ethyl-2-methoxyphenol	4.78–613.76	0.9906	17.80	59.32	7.18	7.29	104.39
4-Hydroxy-3-methoxystyrene	19.04–9747.99	0.9935	441.90	1472.98	4.24	3.72	96.29
4-Methyl-2-methoxyphenol	10.25–2624.53	0.992	50.03	166.77	6.17	4.86	88.98
**Terpenes**							
2-Undecanone	0.57–290.54	0.9921	0.04	0.14	3.26	5.65	101.13
β-Damascenone	0.55–278.98	0.9989	62.90	209.66	3.87	7.23	88.41
Farnesol	6.80–435.15	0.9965	25.24	84.12	4.28	1.32	94.25
α-Cedrene	3.49–1785.27	0.9974	15.24	50.81	6.77	2.81	98.43
Caryophyllene	0.42–108.51	0.9957	22.90	76.33	9.34	5.04	102.94
Rosoxide	0.39–100.35	0.9974	3.16	10.54	4.77	5.21	100.54
Citronellol	0.31–160.93	0.9959	12.64	42.14	1.28	9.19	105.53
Geraniol	0.15–77.84	0.9939	64.43	214.78	2.47	8.60	95.81
Irisone	0.03–17.47	0.9966	15.55	51.84	3.46	1.82	110.83
Geranylacetone	0.51–130.62	0.9912	1.06	3.54	4.73	3.82	98.84
β-Cyclocitral	0.49–251.81	0.9949	14.85	49.50	6.64	6.89	107.52
Cineole	0.72–367.11	0.9983	79.35	264.50	4.29	0.57	103.62
Terpinen-4-ol	0.39–199.60	0.9961	24.68	82.27	2.92	3.64	93.41
Cedrol	1.10–560.10	0.9994	7.49	24.98	4.54	9.51	100.04
Isophorone	0.73–375.64	0.9903	4.70	15.66	4.73	6.36	96.71
Linalool	0.63–320.22	0.999	1.82	6.07	8.36	4.05	100.05
**Lactones**							
γ-Decalactone	0.26–133.12	0.9951	19.67	65.58	4.08	8.01	97.61
γ-Dodecalactone	4.72–604.68	0.99	22.66	75.54	0.14	2.99	102.34
γ-Nonolactone	5.32–2722.23	0.9975	89.68	298.93	4.16	8.72	100.66
γ-Hexalactone	3.53–112.25	0.9909	1.77	5.90	5.31	1.56	103.76

**Table 3:** Concentrations (μg/L) of volatile compounds in three different types of Baijiu.

Volatile Compounds	Odor Thresholds	Fenjiu		Wuliangye		Guotai	
		Concentration	OAV	Concentration	OAV	Concentration	OAV
**Esters**							
Isoamyl acetate	94	5339.58 ± 951.27	56.80	6423.77 ± 922.57	68.34	9176.91 ± 250.18	97.63
Ethyl phenylacetate	407	189.57 ± 26.6	0.47	688.71 ± 32.78	1.69	568.37 ± 27.91	1.40
Phenethyl acetate	909	146.62 ± 32.39	0.16	344.29 ± 36.29	0.38	109.58 ± 8.14	0.12
Phenethyl butyrate	961	1.37 ± 0.2	0.00	47.4 ± 3.52	0.05	20.53 ± 1.15	0.02
Ethyl propionate	19,019	853.43 ± 41.34	0.04	1108.46 ± 181.78	0.06	6932.92 ± 393.8	0.36
Isobutyl hexanoate	5250	6.83 ± 0.05	0.00	316.88 ± 38.23	0.06	24.36 ± 1.51	0.00
Isoamyl butyrate	915	8.21 ± 0.17	0.01	73.86 ± 12.66	0.08	nd	—
Ethyl laurate	500	150.93 ± 50.11	0.30	500.38 ± 53.63	1.00	116.53 ± 4.95	0.23
Butyl butyrate	110	nd	—	nq	—	4.55 ± 0.2	0.04
Ethyl decanoate	1120	441.88 ± 26.09	0.39	2389.6 ± 119.02	2.13	1338.32 ± 81.26	1.19
Diethyl succinate	35,3193	4886.71 ± 38.29	0.01	1129.74 ± 168.32	0.00	1946.71 ± 10.21	0.01
Ethyl nonanoate	3150	79.42 ± 7.04	0.03	429.59 ± 108.49	0.14	363.75 ± 24.23	0.12
Butyl acetate	2.63	6.24 ± 0.65	2.37	14.15 ± 1.61	5.38	22.71 ± 0.48	8.63
Ethyl 3-phenylpropionate	125	61.69 ± 0.73	0.49	905.9 ± 0.26	7.25	98.75 ± 7.94	0.79
Isoamyl octanoate	600	33.51 ± 4.57	0.06	519.45 ± 13.33	0.87	137.92 ± 2.81	0.23
Ethyl 4-methylpentanoate	21.4	62.42 ± 4.35	2.92	622.67 ± 166.24	29.10	263.38 ± 12.52	12.31
Ethyl cyclohexanoate	20.2	1.61 ± 0.07	0.08	23.56 ± 1.15	1.17	3.8 ± 0.01	0.19
Ethyl 2-methylpentanoate	*	nd	—	6.58 ± 1.96	—	2.29 ± 0.26	—
Pentyl hexanoate	14,000	nd	—	269.42 ± 42.46	0.02	20.26 ± 1.45	0.00
Ethyl 3-methylpentanoate	18	1.17 ± 0.02	0.07	nd	—	6.75 ± 0.02	0.38
Propyl hexanoate	12,800	nq	—	1001.98 ± 74.85	0.08	115.16 ± 4.12	0.01
Hexyl hexanoate	1890	21.26 ± 1.79	0.01	407.91 ± 104.9	0.22	141.88 ± 10.43	0.08
Ethyl 2-methylbutyrate	18	241.71 ± 2.61	13.43	3119.13 ± 48.35	173.29	2366.74 ± 49.87	131.49
Methyl benzoate	0.073	2.32 ± 0.04	31.78	3.84 ± 0.16	52.60	3.9 ± 0.17	53.42
Ethyl benzoate	1430	143.21 ± 27.72	0.10	179.29 ± 8.01	0.13	366.95 ± 10.15	0.26
Ethyl 2-methylpropionate	57.47	329.46 ± 20.21	5.73	1464.44 ± 78.06	25.48	2545.15 ± 156.29	44.29
**Alcohols**							
Phenyl alcohol	28,900	3256.13 ± 237.93	0.11	3049.82 ± 12.37	0.11	7868.06 ± 49.55	0.27
Benzyl alcohol	40,900	123.76 ± 8.78	0.00	144.44 ± 5.59	0.00	1145.19 ± 89.22	0.03
Hexanol	5370	5472.49 ± 16.92	1.02	71,913.75 ± 171.52	13.39	10,216.49 ± 234.94	1.90
Heptanol	26,600	262.14 ± 12.37	0.01	1221.84 ± 7.3	0.05	779.65 ± 7.08	0.03
Octanol	1100	70.9 ± 14.14	0.06	261.84 ± 3.25	0.24	684.62 ± 1.55	0.62
Nonanol	50	54.29 ± 10.27	1.09	63.44 ± 0.61	1.27	33.14 ± 0.2	0.66
1-Octen-3-ol	6.12	98.79 ± 4.68	16.14	197.71 ± 3.23	32.31	92.95 ± 5.78	15.19
2-Heptanol	1430	12.62 ± 8	0.01	1273.52 ± 7.73	0.89	391.19 ± 41.99	0.27
3-Octanol	393	nd	—	30.99 ± 2.27	0.08	173.23 ± 4.44	0.44
2-Nonanol	75	43.4 ± 4.37	0.58	69.37 ± 15.35	0.92	41.14 ± 2.25	0.55
Pentanol	4000	562.71 ± 7.22	0.14	1556.27 ± 537.43	0.39	1462.39 ± 176.97	0.37
**Acids**							
Pentanoic acid	389	349.62 ± 10.85	0.90	6631.68 ± 1004.73	17.05	3336.22 ± 318.74	8.58
Heptanoic acid	13,300	1024.96 ± 86.08	0.08	17,513.91 ± 569.26	1.32	5493.77 ± 75.02	0.41
Octanoic acid	2700	918.51 ± 97.38	0.34	6287.55 ± 704.09	2.33	4896.17 ± 11.55	1.81
Decanoic acid	500	414.33 ± 41.32	0.83	2248.13 ± 52.02	4.50	864.62 ± 3.32	1.73
3-Methylbutanoic acid	1050	970.4 ± 40.16	0.92	6946.24 ± 76.03	6.62	2629.44 ± 181.79	2.50
4-Methylpentanoic acid	144	147.63 ± 15.04	1.03	1028.92 ± 293.65	7.15	766.3 ± 58.65	5.32
**Aldehydes and ketones**							
Decanal	12	61.26 ± 0.78	5.11	581.48 ± 13.79	48.46	316.29 ± 66.91	26.36
(*E*)-2-Heptenal	0.0046	18.56 ± 0.08	4034.78	2.45 ± 0.07	532.61	20.53 ± 0.75	4463.04
(*E*)-2-Nonenal	51	16.58 ± 0.47	0.33	71.87 ± 8.2	1.41	51.29 ± 12.93	1.01
(*E*)-2-Octenal	*	nd	—	26.98 ± 8.16	—	58.71 ± 1.83	—
2,3-Butanedione	5	nd	—	nd	—	877.35 ± 18.33	175.47
3-Hydroxy-2-butanone	259	2102.04 ± 1637.78	8.12	15,734.59 ± 322.84	60.75	52,264.56 ± 487.69	201.79
2-Methylpropanal	1300	657.74 ± 16.75	0.51	3540.37 ± 620.42	2.72	7700.86 ± 884.05	5.92
2-Methylbutanal	16	161.22 ± 6.82	10.08	535.09 ± 14.85	33.44	887.15 ± 9.04	55.45
Benzaldehyde	4200	496.91 ± 33.56	0.12	1102.29 ± 94.87	0.26	945.66 ± 198.82	0.23
2-Octanone	50	47.32 ± 11.86	0.95	276.17 ± 52.66	5.52	217.12 ± 7.27	4.34
Phenylacetaldehyde	262	4799.96 ± 240.22	18.32	6189.03 ± 1958.57	23.62	2808.12 ± 95.44	10.72
Propanal	2	544.13 ± 22.67	272.07	1021.4 ± 143.35	510.70	4236.21 ± 270.52	2118.11
Octanal	40	30.54 ± 5.51	0.76	128.18 ± 2.58	3.20	73.92 ± 3.71	1.85
Nonanal	122	285.32 ± 10.39	2.34	734.07 ± 60.83	6.02	497.54 ± 28.48	4.08
1-Octen-3-one	0.05	3.55 ± 0.1	71.00	6.13 ± 0.24	122.60	3.28 ± 0.06	65.60
(*E*,*Z*)-2,6-Nonadienal	0.64	4.87 ± 0.19	7.61	7.31 ± 1.03	11.42	5.25 ± 0.31	8.20
3-Methylbutanal	17	14,920.17 ± 419.99	877.66	58,465.44 ± 290.21	3439.14	82,260.03 ± 392.72	4838.83
(*E*,*E*)-2,4-Hexadienal	*	nd	—	9.87 ± 0.07	—	nd	—
(*E*,*E*)-2,4-Decadienal	7.71	12.93 ± 1.17	1.68	14.63 ± 0.15	1.90	13.27 ± 0.38	1.72
(*E*,*E*)-2,4-Nonadienal	0.0026	3.46 ± 0.36	1330.77	32.2 ± 3.75	12384.62	11.19 ± 0.1	4303.85
Hexanal	25.5	151.24 ± 24.36	5.93	845.76 ± 11.03	33.17	640.99 ± 28.31	25.14
1,1,3-Triethoxypropane	3700	283.61 ± 30.7	0.08	nd	—	306.05 ± 0.46	0.08
2-Nonanone	483	13.77 ± 0.49	0.03	219 ± 16.22	0.45	179.96 ± 8.08	0.37
Acetophenone	256	12.38 ± 3.7	0.05	119.71 ± 40.44	0.47	186.4 ± 0.47	0.73
**Furans**							
5-Methyl-2-acetylfuran	40,900	45.89 ± 1.09	0.00	155.35 ± 4.14	0.00	279.74 ± 7.28	0.01
Furfuryl alcohol	2000	77.07 ± 1.73	0.04	1252.52 ± 378.8	0.63	4574.64 ± 4.98	2.29
Furfural	44,000	9185.87 ± 87.24	0.21	26,655.25 ± 1198.7	0.61	38,657.5 ± 3092.53	0.88
2-Acetylfuran	58,504	42.42 ± 1.81	0.00	548.31 ± 43.39	0.01	2031.18 ± 228.43	0.03
Ethyl 2-furoate	130,000	40.55 ± 1.5	0.00	499.12 ± 63.31	0.00	222.37 ± 21.18	0.00
5-Methyl furfural	466,000	41.91 ± 3.79	0.00	677.38 ± 95.46	0.00	2484.69 ± 33.48	0.01
**Pyrazines**							
2,6-Dimethylpyrazine	791	nq	—	456.93 ± 106.52	0.58	2352.68 ± 62.85	2.97
2-Methylpyrazine	121927	44.59 ± 3.97	0.00	69.84 ± 11.73	0.00	470.1 ± 5.31	0.00
2,3,5,6-Tetramethylpyrazine	80,100	35.58 ± 1.91	0.00	122.35 ± 5.47	0.00	1191.78 ± 89.18	0.01
2-Methyl-6-ethylpyrazine	40	8.14 ± 0.06	0.20	97.83 ± 6.67	2.45	928.97 ± 5.64	23.22
2,3,5-Trimethylpyrazine	730	24.58 ± 5.77	0.03	151.92 ± 1.95	0.21	578.88 ± 18.35	0.79
2,3-Diethylpyrazine	172	nd	—	nd	—	7.12 ± 0.17	0.04
2,3-Diethyl-5-methylpyrazine	*	nd	—	3.93 ± 0.1	—	3.89 ± 0.71	—
2,3-Dimethylpyrazine	10,824	10.9 ± 4.25	0.00	5.48 ± 2.54	0.00	84.91 ± 4.12	0.01
**Sulfur compounds**							
Methional	7.12	nd	—	nd	—	73.68 ± 14.19	10.35
Methyl furfuryl disulfide	0.4	nd	—	nd	—	10.14 ± 0.2	25.35
Dimethyl disulfide	9	23.77 ± 0.29	2.64	97.71 ± 6.11	10.86	121.77 ± 7.55	13.53
Furfuryl mercaptan	0.1	11.24 ± 0.73	112.40	nd	—	35.2 ± 1.01	352.00
Ethyl 3-methylthiopropionate	3080	nd	—	nd	—	48.41 ± 0.75	0.02
Methyl thiobutyrate	0.14	nd	—	113.15 ± 21.92	808.21	14.15 ± 0.36	101.07
Thiazole	740	38.08 ± 6.53	0.05	42.79 ± 0.13	0.06	85.21 ± 7.69	0.12
Dimethyl trisulfide	0.36	43.97 ± 5.01	122.14	172.73 ± 2.22	479.81	182.28 ± 6.62	506.33
Methionol	2110	nd	—	nd	—	732.65 ± 24.54	0.35
Methyl 2-methyl-3-furyl disulfide	0.02	0.44 ± 0.04	22.00	0.56 ± 0.06	28.00	0.94 ± 0.09	47.00
Methanethiol	2	185.05 ± 27.02	92.53	238.18 ± 26.04	119.09	249.95 ± 4.03	124.98
**Phenols**							
4-Methylphenol	167	28.16 ± 0.91	0.17	1530.14 ± 8.94	9.16	127.14 ± 13.81	0.76
3-Methylphenol	*	nq	—	1.38 ± 0.17	—	9.61 ± 0.01	—
Phenol	18,900	73.34 ± 5.76	0.00	539.69 ± 13.9	0.03	235.54 ± 6.55	0.01
4-Ethylphenol	123	54.51 ± 5.87	0.44	322.84 ± 5.13	2.62	72.88 ± 3.43	0.59
4-Ethyl-2-methoxyphenol	123	108.8 ± 6.39	0.88	161.72 ± 2.08	1.31	nd	—
4-Hydroxy-3-methoxystyrene	209	21.47 ± 0.03	0.10	nd	—	25.98 ± 4.19	0.12
4-Methyl-2-methoxyphenol	315	6.64 ± 0.18	0.02	43.97 ± 1.04	0.14	82.63 ± 1.1	0.26
**Terpenes**							
2-Undecanone	6	nq	—	12.67 ± 1.98	2.11	9.19 ± 4.05	1.53
β-Damascenone	0.12	6.55 ± 1.71	54.58	9.93 ± 0.06	82.75	8.69 ± 0.95	72.42
Farnesol	*	nd	—	14.87 ± 1.22	—	20.04 ± 0.86	—
α-Cedrene	6500	5.88 ± 0.83	0.00	8.86 ± 0.2	0.00	6.82 ± 0.03	0.00
Caryophyllene	130	1.68 ± 0.24	0.01	4.75 ± 0.36	0.04	21.74 ± 0.14	0.17
Rosoxide	*	1.57 ± 0.09	—	nd	—	nd	—
Citronellol	300	4.34 ± 0.59	0.01	3.42 ± 0.27	0.01	6.22 ± 0.8	0.02
Geraniol	120	8.72 ± 0.75	0.07	4.12 ± 0.05	0.03	10.85 ± 0.31	0.09
Irisone	1.3	0.59 ± 0.01	0.45	0.69 ± 0.01	0.53	1.11 ± 0.04	0.85
Geranylacetone	60	11.82 ± 0.9	0.20	23.66 ± 3.94	0.39	46.04 ± 7.22	0.77
β-Cyclocitral	3	4.3 ± 1	1.43	9.45 ± 0.73	3.15	2.11 ± 0	0.70
Cineole	0.55	5.09 ± 0.55	9.25	nd	—	35.48 ± 4.06	64.51
Terpinen-4-ol	940	3.66 ± 0.13	0.00	3.48 ± 0.38	0.00	2.93 ± 0.11	0.00
Cedrol	7300	3.41 ± 1.77	0.00	19.99 ± 3.25	0.00	54.48 ± 1.88	0.01
Isophorone	11	18.09 ± 1.15	1.64	10.74 ± 3.55	0.98	14.52 ± 0.69	1.32
Linalool	13.1	48.07 ± 1.94	3.67	45.33 ± 6.54	3.46	77.84 ± 3.48	5.94
**Lactones**							
γ-Decalactone	11	3.21 ± 1.01	0.29	0.28 ± 0.03	0.03	9.18 ± 0.37	0.83
γ-Dodecalactone	60.68	24.56 ± 3.45	0.40	42.16 ± 6.61	0.69	36.92 ± 1.27	0.61
γ-Nonolactone	91	121.36 ± 1.77	1.33	198.03 ± 2.78	2.18	275.61 ± 6.94	3.03
γ-Hexalactone	359,000	47.23 ± 1.04	0.00	54.51 ± 20.82	0.00	nd	—

*: The odor threshold of the compound has not been determined or obtained from literature. nd: The compound has not been detected in this sample. nq: The compound has not been quantified in this sample.

## Data Availability

The data that support the findings of this study are available from the corresponding author upon request.

## Sample Availability

Samples are available from the corresponding authors.
